# Supplementary material for: European Robin Cryptochrome-4a Associates with Lipid Bilayers in an Ordered Manner, Fulfilling a Molecular-Level Condition for Magnetoreception
Source: ACS Chem Biol. 2025 Feb 21;20(3):592–606. doi: 10.1021/acschembio.4c00576 (PMC11934094; doi:10.1021/acschembio.4c00576)
Supplement: Supplementary file 4 — cb4c00576_si_004.pdf [file cb4c00576_si_004.pdf]

European Robin Cryptochrome-4a Associates with Lipid Bilayers in an Ordered Manner, Fulfilling a Molecular-Level Condition for Magnetoreception
(Supporting Information)

Marta Majewska[1†], Maja Hanić[2†], Rabea Bartölke[3†], Jessica Schmidt[3], Justyna Bożek[1], Luca Gerhards[2], Henrik Mouritsen[3,4], Karl-Wilhelm Koch[4,5], Ilia A. Solov'yov[2,4,6]* and Izabella Brand[1,4#]

[1] Institute of Chemistry, School of Mathematics and Science, Carl von Ossietzky Universität Oldenburg, 26111 Oldenburg, Germany

[2] Institute of Physics, School of Mathematics and Science, Carl von Ossietzky Universität Oldenburg, 26111 Oldenburg, Germany

[3] Animal Navigation, Institute of Biology and Environmental Sciences, School of Mathematics and Science, Carl von Ossietzky Universität Oldenburg, D-26111 Oldenburg, Germany

[4] Research Center for Neurosensory Sciences, Carl von Ossietzky Universität Oldenburg, D-26111 Oldenburg, Germany

[5] Division of Biochemistry, Department of Neuroscience, Carl von Ossietzky Universität Oldenburg, D-26111 Oldenburg, Germany

[6] Institute of Physics, Center for Nanoscale Dynamics (CENAD), Carl von Ossietzky Universität Oldenburg, 26129 Oldenburg, Germany

* Ilia A. Solov'yov

Email: ilia.solovyov@uni-oldenburg.de

Izabella Brand

Email: izabella.brand@uni-oldenburg.de

† These authors contributed equally

S1. Composition of the model membrane of the outer segment cone of the vertebrate photoreceptor cell

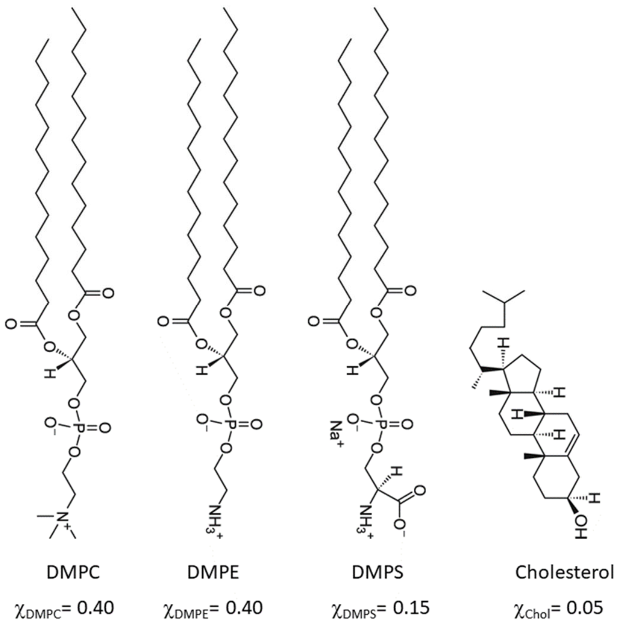

Figure S1. The structures and mole fractions of the lipids used to prepare the model membrane of the outer segment of the double cone photoreceptor cell.

Phosphatidylcholine (PC), phosphatidylethanolamine (PE), phosphatidylserine (PS), and cholesterol (chol) compose the outer cell segment of the vertebrate double cone photoreceptor cell.[1-3] A substantial fraction of phospholipids in the outer segment membranes of the cone photoreceptor cells contains long (C14 – C22) mono- and polyunsaturated acyl chains that maintain the membrane in a liquid-disordered state. Stable phospholipids with two saturated myristoyl chains were used to construct a model membrane (see Fig. S1). The liquid state of the lipid mixture was ensured by choosing phospholipids with two relatively short myristoyl (C14) acyl chains. The mole fraction (χ) of each lipid used to prepare the model membrane corresponds to the physiological concentration of the lipids in the outer segment of the vertebrate cone photoreceptor cell and is marked in Fig. S1.

S2. Preparation of the floating membrane on the gold surface

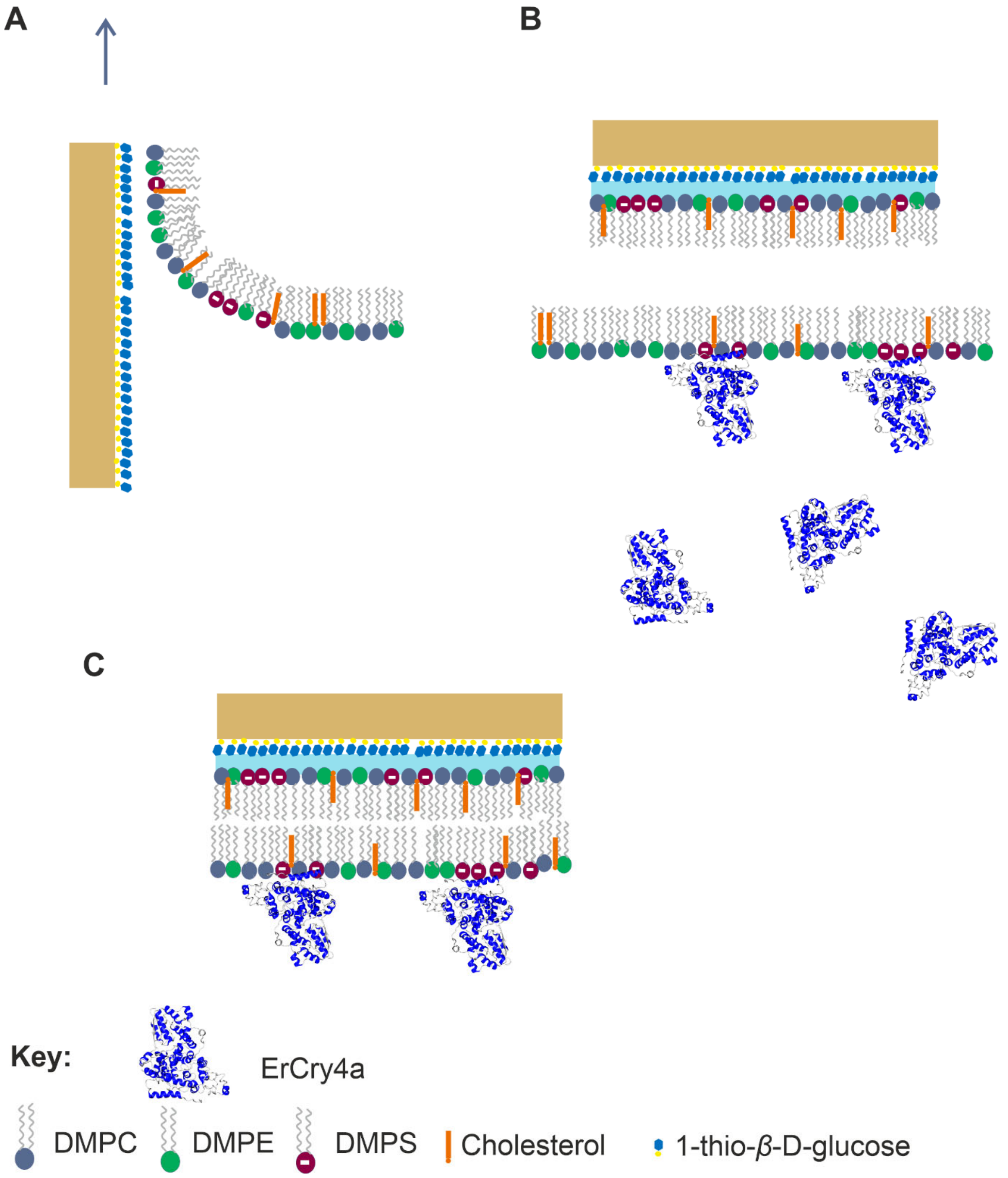

Figure S2. Schematic illustration of the preparation process of a floating model membrane with ErCry4a in the 1-thio-β-D-glucose modified Au surface: **A**: Langmuir-Blodgett vertical withdrawing, **B**: Langmuir-Schaefer transfer **C**: molecular scale order in the model lipid membrane. Blue filling shows water layer separating the thioglucose monolayer from the membrane.

Figure S2 illustrates the principal steps of the preparation process of the model membrane of the outer segment of the double-cone photoreceptor cell.

S3. Equilibrium spreading pressure of ErCry4a

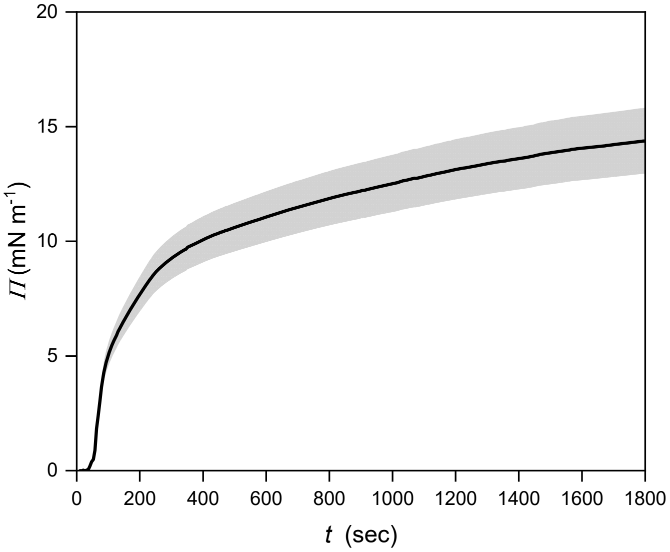

Figure S3. Time dependence of the surface pressure recorded for 100 nM ErCry4a dissolved in 25 mM Tris, 100 mM NaCl, 5 mM $MgCl_2$ electrolyte solution and adsorbed at the air|electrolyte solution interface at 21 °C. Gray line show the error of the measurement, the black curve shows the average curve from five independent measurements.

Figure S3 shows the changes in the surface pressure over time during the accumulation of ErCry4a at the air|water interface. ErCry4a solution, after rebuffering, was injected into the small Langmuir trough and left for possible adsorption at the air|electrolyte interface. The ability of ErCry4a to accumulate at the interface was examined. In all cases, the surface pressure vs time curves displayed two kinetic regimes. In the first kinetic regime the surface pressure increased rapidly shortly after injection of the protein into the solution. In the second regime the growth of the surface pressure over time slow down reaching a plateau value of 13 ± 2 mN m^{-1}. This characteristic is typical for a fast accumulation of the ErCry4a at the air|electrolyte interface followed by slower reorientation of the protein in the monolayer film.[4]

S4. Langmuir isotherms of the lipid mixtures

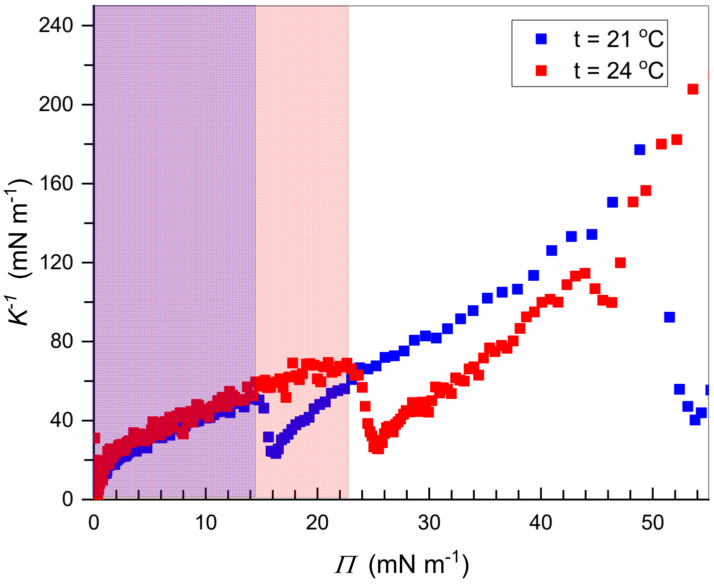

Figure S4. Dependence of compressibility modulus on the surface pressure for the DMPC:DMPE.DMPS:chol monolayers at the air| electrolyte interface recorded at 21 °C (blue) and 24 °C (red). Electrolyte composition includes 100 mM NaCl, 25 mM TRIS and 5 mM $MaCl_2$. The colored blocks show the surface pressure regions where the liquid-disordered phase at 21 °C (blue) and 24 °C (red) are expected.

The compressibility modulus versus surface pressure plots of the PC:PE:PS:chol lipid mixture in the 0.4:0.4:0.15:0.05 mole ratio at the air|electrolyte interface are shown in Fig. S4. The compressibility modulus (K^{-1}) is defined as

$$K^{-1} = \left[-\frac{1}{A_\mathrm{m}} \left(\frac{\partial A_\mathrm{m}}{\partial \Pi} \right)_{T,P,ni} \right]^{-1}, \tag{S1}$$

where A_m is the area per molecule in the Langmuir monolayer and Π is the surface pressure. The K^{-1} reflects the physical state of the hydrocarbon chain fragment in an amphiphilic molecule forming the monolayer, see Fig. S3. The K^{-1} indicates that at 21 °C the lipid monolayer exists in a liquid disordered state for Π< 13 mN m^{-1} while at 24 °C for Π< 22 mN m^{-1}. In the liquid-disordered state, the average area per lipid molecule ranges between 0.8 and 0.6 nm^2. At higher surface pressure values, the lipid monolayer exists in a liquid-ordered state, and the average area per molecule drops to 0.5 – 0.4 nm^2. The monolayers collapse at the limiting area per molecule of 0.39 nm^2.

S5. Association of ErCry4a to a DMPC monolayer at the air|electrolyte solution interface

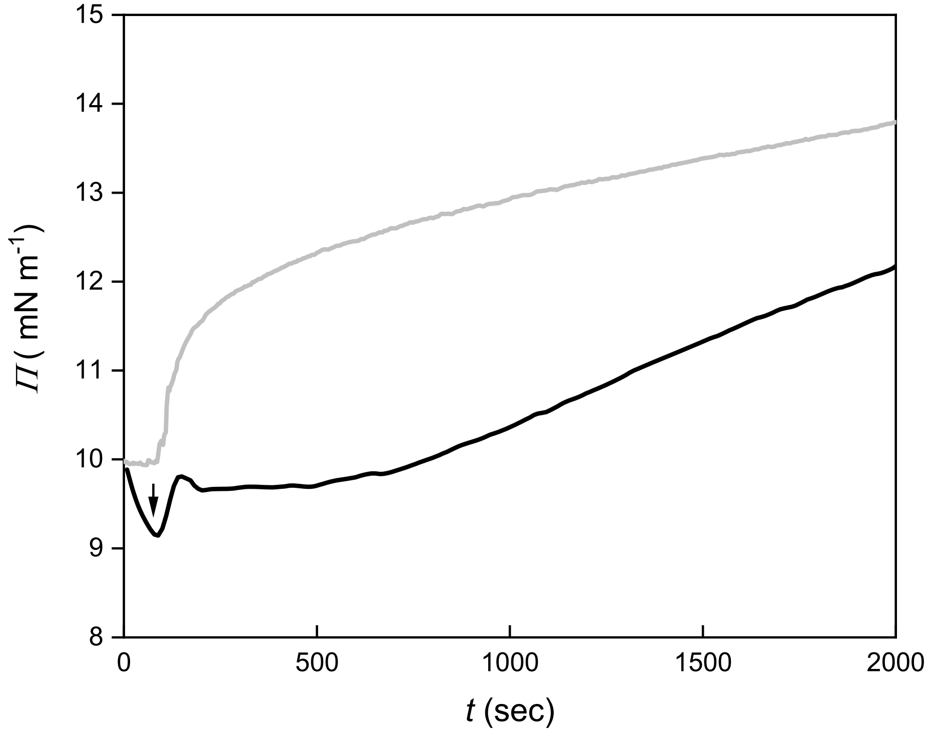

Figure S5. Time dependence of the surface pressure recorded for the DMPC monolayer (black line) and model outer segment lipid monolayer (DMPC:DMPE:DMPS:cholesterol) (gray line), compressed to the surface pressure of 10 mN m^{-1}, after injection of 100 nM ErCry4a (black arrow). Electrolyte solution contained 25 mM Tris, 100 mM NaCl and 5 mM $MgCl_2$ in H_2O, temperature 24 °C.

Figure S5 shows that the model outer segment lipid monolayer and DMPC monolayer interact differently with ErCry4a. ErCry4a is able to interact with the DMPC monolayer after ca. 600 s lag-time. The surface pressure increases slowly over the longer time of interaction (up to 2000 s). This result suggests that the protein approaching the monolayer surface undergoes some reorientation, or even conformational changes before it is associated with the lipid monolayer. In contrast, ErCry4a displays a fast adsorption and accumulation on the surface of the model outer segment monolayer, as proved by a fast increase in the surface pressure immediately after injection of the protein.

S6. PM IRRA spectra of the amide I' mode spectral region of ErCry4a in a membrane associated state

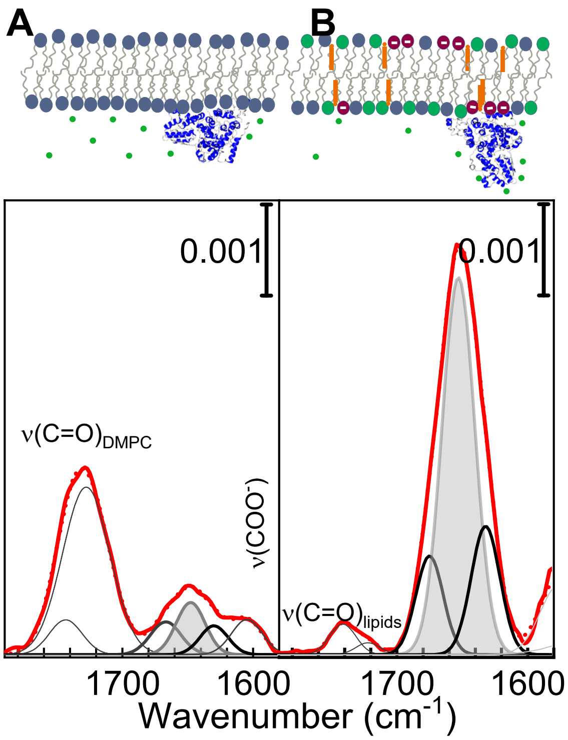

Figure S6. Deconvoluted PM IRRA spectra of the **A:** DMPC and **B:** DMPC:DMPE:DMPS:cholesterol model outer segment membrane and associated ErCry4a deposited on the Au surface at E = 0.0V vs Ag|AgCl reference electrode at 24 °C (red thick curve); Electrolyte solution contained 25 mM d_{11}-Tris, 10 mM NaCl and 5 mM $MgCl_2$ in D_2O. Spectra are shown in the 1780-1580 cm^{-1} region. Thin black and gray lines show the results of deconvolution while the shaded area corresponds to the amide I' band of the α-helices. Insets illustrate the experimental conditions: Scale bars correspond to absorbance values measured in arbitrary units. Upper panel: illustration of the lipid membrane with attached ErCry4a, green dots represent Mg^{2+} ions.

S7. Secondary structure elements of ErCry4a in solution and the membrane-associated state

Infrared spectroscopy is an excellent method to determine the secondary structure of proteins *in situ*. The amide I vibration mode appears in proteins between 1700 cm^{-1} and 1600 cm^{-1} and originates predominantly from the ν(C=O) stretching mode (ca. 76%) with contributions from the ν(CN) stretching mode and in-plane bending of the NH groups.[5, 6] To avoid the overlap of the amide I band with the OH deformation mode in water (appearing usually in the 1660−1620 cm^{-1} range), the IR spectra of proteins are often measured in D_2O; in this case, the nomenclature changes to amide I' mode. D_2O does not cause a significant spectral shift of the amide I' vibration mode. Different secondary structure elements in proteins: α-helices, parallel and antiparallel β-sheets, β-turns, or random coils give the amide I' mode at different characteristic frequencies.[5-7] Therefore, the deconvolution of the amide I' mode makes possible a qualitative and quantitative (%) assignment of different secondary structure elements in proteins. Table S1 shows the assignment of the secondary structure elements in ErCry4a in aqueous solution and membrane-associated state.

Table S1. The wavenumber of the absorption maxima of the deconvoluted amide I' vibration modes in ErCry4a in aqueous solution and in membrane associated state at the following environmental conditions: at 21 °C and 24 °C in electrolyte solution containing 25 mM d_{11}-Tris, 100 mM NaCl and 5 mM $MgCl_2$ in D_2O as well as at 24 °C in electrolyte solution containing 25 mM d_{11}-Tris, 100 mM NaCl in D_2O.

**Environment**	**Wavenumber (cm^{-1}) and relative intensity of the deconvoluted amide I' band in ErCry4a assigned to the secondary structure elements**					
	**β-sheet**	**Other elem. (turns)**	**α-helix**	**Other elem. (coil)**	**β-sheet**	**Agg. β-sheet**
Solution<br>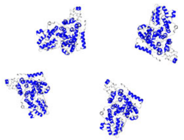	1689 vw	1675 m	1653 s	1639 vw	1623 m	
OS Membrane;<br>21°C, 5mM Mg^{2+}<br>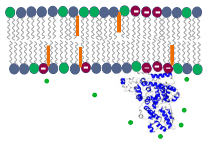		1666 vw<br>(unfolded, misfolded)			1624vw	
OS Membrane;<br>24°C, 5mM Mg^{2+}<br>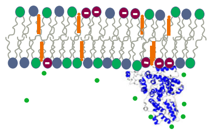		1670 m	1650 vs		1623 m	
OS Membrane;<br>24°C<br>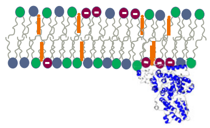		1670 m	1650 vs		1623 m	1614 w
DMPC bilayer,<br>24°C<br>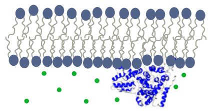		1666 m	1647 m		1623 w	

Relative intensities of the amide I' band components: vw – very weak; w – weak, m – medium; s -strong, vs – very strong; Abbreviations: other elem. – other elements, Agg. - aggregated

S8. Quantitative analysis of the amide I' mode of the model otter segment membrane-associated ErCry4a

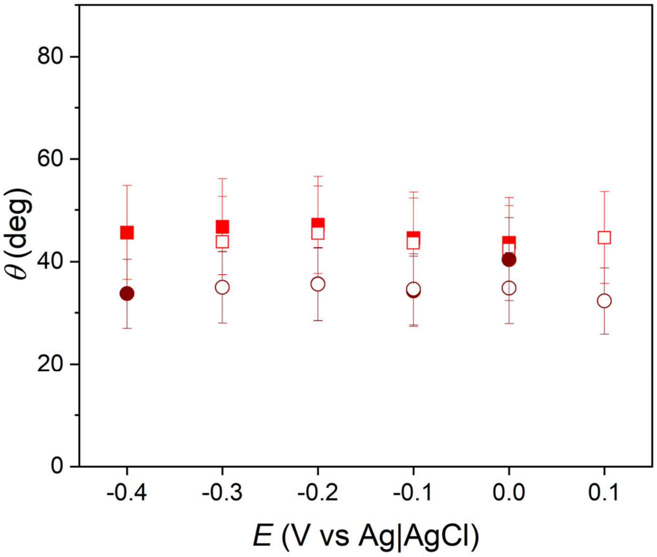

Figure S7. Potential dependence of the average angle between the transition dipole moment vectors of the amide I' vibration modes of the α-helices in the membrane-bound ErCry4a and the surface normal. Recordings were done at 24 °C in the presence of 5 mM $MgCl_2$ (red squares) and in the absence of Mg^{2+} (dark red circles); Electrolyte solution contained 25 mM d_{11}-Tris, 100 mM NaCl in D_2O electrolyte solution; opened symbols: positive and filled symbols: negative going potential scan.

The amide I' mode of membrane-associated ErCry4a was deconvoluted, and the integral intensities as well as and the percent content of the α-helices was determined. In the solution phase the content of α-helices equaled 50 ± 2 %. Optical constants were calculated from the solution spectrum and were used to calculate an IR spectrum of randomly distributed ErCry4a in a film of a thickness of a protein monolayer with the coverage varying between 100% and 70 %. In an anisotropic film, some IR absorption modes may be enhanced while others attenuated.[8] The integral intensity ($\int Ad\nu$) of a measured IR absorption mode depends on the relative orientation of the electric field vector of the reflected electromagnetic radiation (normal to the surface) and the direction of the transition dipole moment vectors:

$$\int \mathrm{A}d\nu \propto \Gamma\left|\vec{\mu}\cdot\vec{E}\right|^2 = \Gamma|\vec{\mu}|^2\left|\vec{E}\right|^2\cos^2\theta \qquad \text{(S2)},$$

where θ is the is the angle between $\vec{\mu}$ and $\vec{E}$ vectors, and Γ is the surface concentration of the species covering the reflecting IR radiation surface (gold).

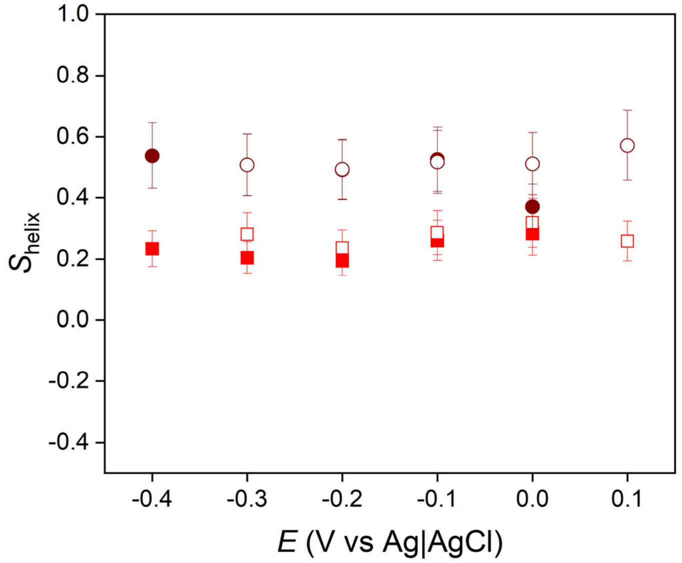

Figure S8. Potential dependence of the order parameter of the transition dipole moment vector of the of the amide I' mode of α-helices (S_{helix}) in membrane bound ErCry4a and surface normal versus. The values correspond to the measurements at 24 °C in the presence of 5 mM $MgCl_2$ (red squares) and in its absence (dark red circles); Electrolyte solution contained 25 mM d_{11}-Tris, 100 mM NaCl in D_2O electrolyte solution; opened symbols: positive- and filled symbols: negative-going potential scan.

The integral intensities of the amide I' vibrational modes were used to determine the average angle θ between the transition dipole moments of the amide I' vibrational modes arising in all 26 helices in ErCry4a and the surface normal (direction of the electric field of the reflected IR beam). This calculation was done as a function of the electric potential applied to the gold electrode. The results are shown in Fig. S7.

The order parameter values of the α-helices (S_{helix}) in the ErCry4a bound to the model membrane are shown in Fig. S8. These values differ from 0, indicating an anisotropic orientation of the membrane associated ErCry4a.

S9. Correlation between the intensity of an IR absorption band in IRRAS and the orientation of a molecule in an anisotropic film

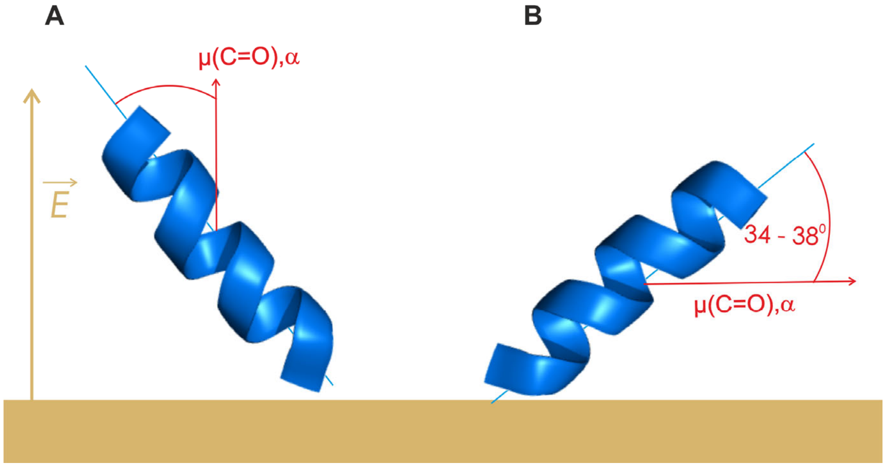

Figure S9. Limiting cases for the orientation of an α-helical fragment in a protein adsorbed on a solid surface; **A**: parallel and **B**: normal orientation of the transition dipole vector versus the electric field vector. A blue line shows the direction of the long axis of the α-helix. The direction of the transition dipole vector of the amide I' mode (red arrow) and the direction of the electric field vector of the *p*-polarized light at the phase boundary (gold arrow) are indicated.

Depending on the value of θ, and thus on the orientation of a molecule in the studied film, some IR absorption bands may be enhanced while others may disappear from the IRRA spectrum.[8] Figure S7 shows two limiting cases for the orientation of an α-helical fragment in a protein adsorbed on a solid surface leading to the enhancement and cancellation of the amide I' vibration mode in the IRRA spectrum. A parallel orientation of $\vec{\mu}$ and $\vec{E}$ vectors cause their strong coupling resulting in the enhancement of the IR absorption band of the amide I' mode, see Fig. S9A. Once the angle between $\vec{\mu}$ and $\vec{E}$ equals 90º, see Fig. S9B, according to Eq. (S2), the integral intensity of the amide I' mode decreases to zero. In this case there is no coupling of the transition dipole and the electric field vectors.

S10. Determination of the angle between the transition dipole vector of a given IR absorption band and normal to the membrane surface based on molecular dynamics simulations

The tilt angle of the helices in lipid bilayer associated ErCry4a with respect to the membrane surface was determined according to procedure described in our previous publication.[9] Briefly, each amide group of the protein includes an IR-active C=O bond aligning with the corresponding transition dipole moment. The dipole moment for each amino acid is characterized by a tilt angle θ_n, computed relatively to the membrane surface normal as

$$\theta_n = \arccos\left(\frac{\overrightarrow{d_n}\vec{z}}{\left|\overrightarrow{d_n}\right|\left|\vec{z}\right|}\right), \qquad \text{(S3)}$$

where $\vec{d}_n$ is the transition dipole moment of the C=O stretching mode (ν(C=O) mode) in the n-th residue and $\vec{z}$ is the normal vector pointing perpendicular to the membrane surface. The resulting time average angle $\langle\theta\rangle$ for the whole melittin can be computed as:

$$\langle\theta\rangle = \frac{\sum_{n=1}^{N}\theta_n w_n}{\sum_{n=1}^{N} w_n}, \qquad \text{(S4)}$$

where the weights w_n describe the coupling of the transition dipole moments of the ν(C=O) modes in melittin to the electric field vector of the reflected IR radiation and are defined as the z-component of the normalized vector of the respective dipole moment $w_n = \left|\overrightarrow{d_n}\right|_z$.

S11.Physical characteristics of the simulated model membrane

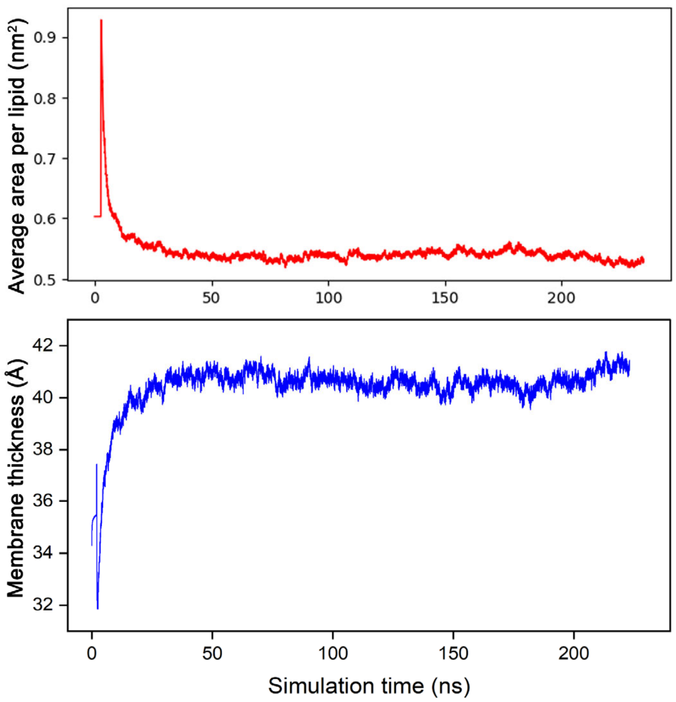

Figure S10. Upper panel: Time-dependence of the average area per lipid plotted for the model membrane employed in MD simulations. Lower panel: Time-dependence of the membrane thickness.

S12. Primary structure analysis of the ErCry4a protein

Table S2. Compositional table of the ErCry4a at the primary structure level. The analysis was performed with the use of the SAPS software.[10] Occurrence probability shows the number of residues for a certain amino acid appearing in the protein.

Amino acid	Occurrence count (Frequency, %)	Amino acid	Occurrence count (Frequency, %)
A	33 (6.3)	D	26 (4.9)
G	27 (5.1)	I	28 (5.3)
M	13 (2.5)	P	30 (5.7)
S	33 (6.3)	V	21 (4.0)
C	11 (2.1)	E	36 (6.8)
H	21 (4.0)	K	22 (4.2)
N	17 (3.2)	Q	28 (5.3)
T	29 (5.5)	W	14 (2.7)
F	23 (4.4)	R	32 (6.1)
L	64 (12.1)	Y	19 (3.6)

S13. Distance analysis between the ErCry4a protein and the model membrane

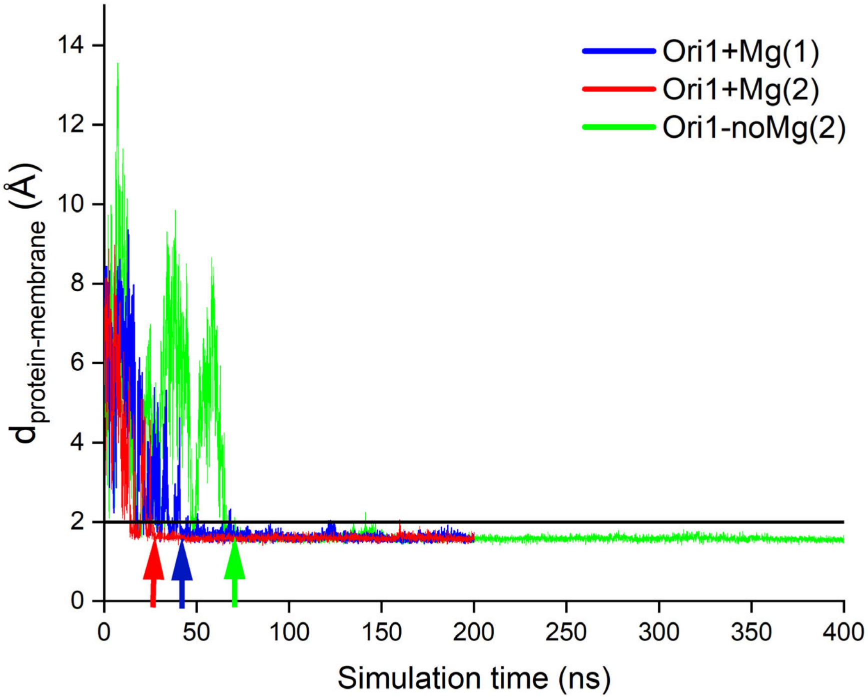

Figure S11. Closest approach analysis performed for different simulations where an interaction with the membrane occurred. The shown distances correspond to the measured distance of any closest atom of the ErCry4a protein to any atom of the model membrane. In corresponding colors, the arrows indicate the moment from which the interaction of the protein and the membrane is considered stable.

S14. Interaction energy analysis between Mg^{2+} ions, the model membrane, and the ErCry4a protein

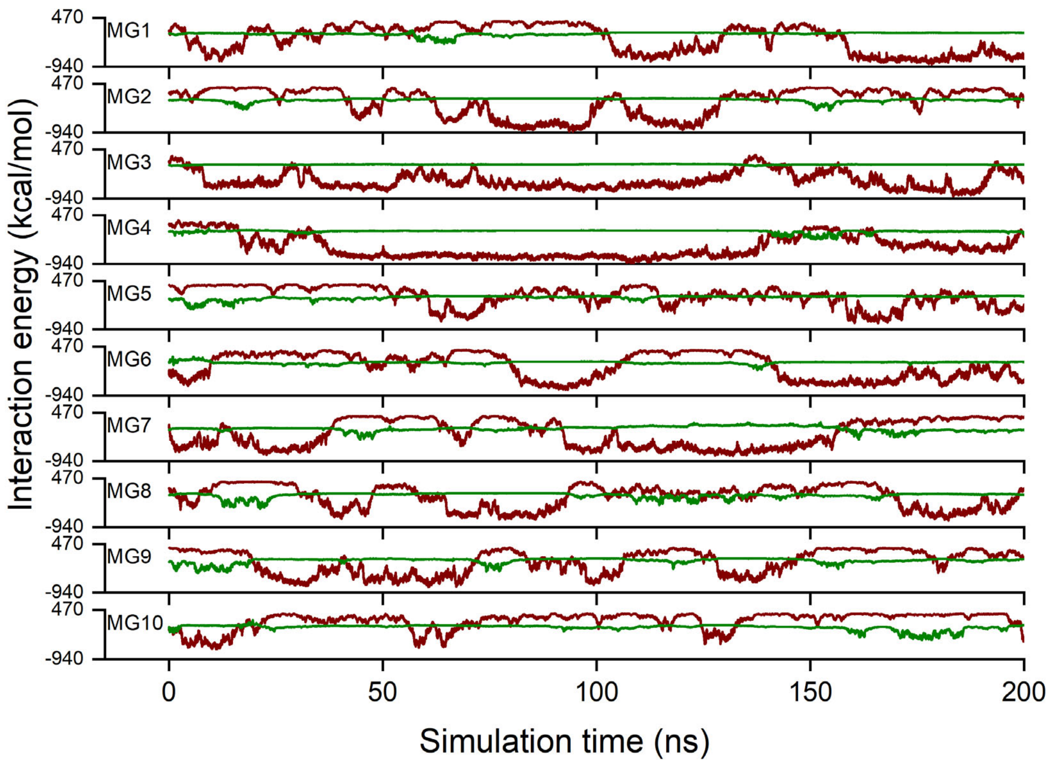

Figure S12. Interaction energies between ErCry4a and the model membrane with every Mg^{2+} ion present in the simulated ErCry4a-membrane system. In this calculation the C-terminal was considered close to the membrane [Ori1+Mg(1)]. Dark red/ green show the interaction energies between Mg^{2+} ions (labeled) and the membrane/ErCry4a, respectively.

S15. Timeline analysis of ErCry4a protein secondary structure changes

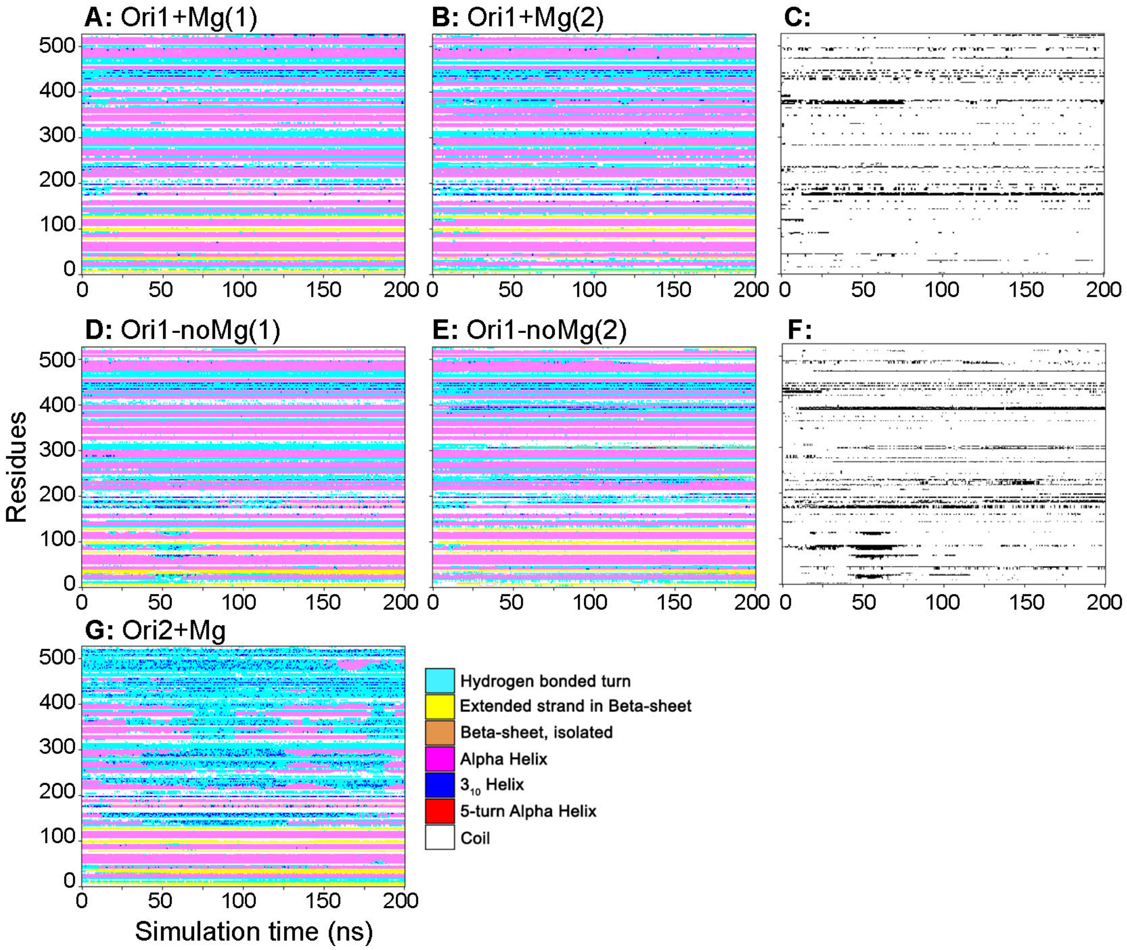

Figure S13. Timeline of the secondary structure analysis of ErCry4a in different performed MD simulations. Panels: **A**, **B**, **D**, **E** and **G** (in color) correspond to the results of the labeled simulations. Panels **C** and **F** (b/w) show the difference between secondary structure panels in their corresponding row. The black color in panels C and F denote the points in which the panels in that row differentiate. The analysis was performed with the VMD and the implemented STRIDE function.

S16. Analysis of ErCry4a secondary structure changes

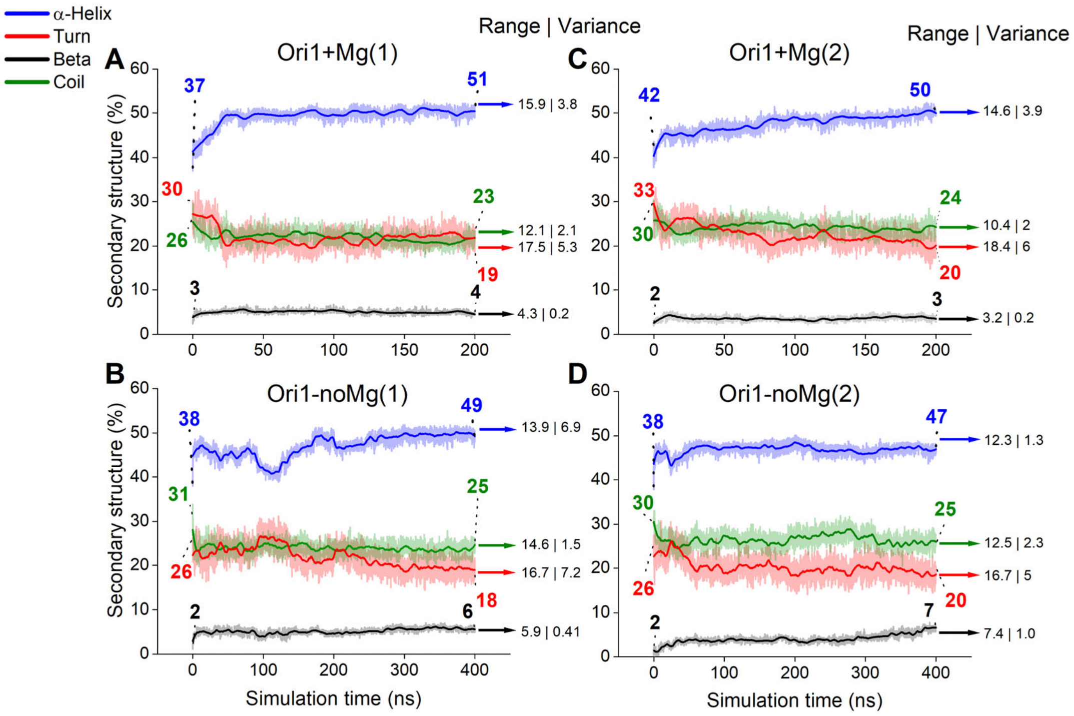

Figure S14. Secondary structure analysis of the ErCry4a protein in the different simulated protein-membrane systems. Time evolution of the secondary structure changes are shown per secondary structure element (distinguished by color) in percentages. The analysis was performed with the VMD and the implemented STRIDE function. The values for the initial and final secondary structure fractions at the end of the simulations are indicated with numbers put next to the corresponding dependencies.

S17. Secondary structure changes of the C-terminal tail in ErCry4a upon interaction with the model membrane

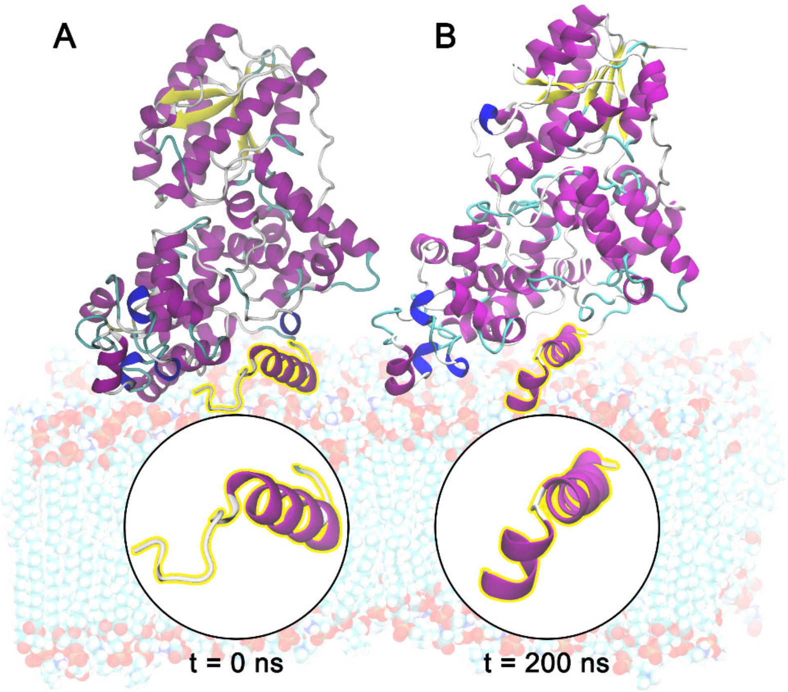

Figure S15. ErCry4a protein colored by secondary structure elements, shown with the C-terminal tail highlighted in yellow. The membrane is shown in the background. **A**: The protein C-terminal tail is unfolded at the beginning of the Ori1+Mg(1) simulation (t = 0 ns) and **B**: At the end of the Ori1+Mg(1) simulation (t = 200 ns) the secondary structure of the C-terminal changes. Here the association of the protein with the membrane occurred, and the C-terminal tail has folded into an α-helix.

S18. Hydrogen bonds between the model membrane and ErCry4a

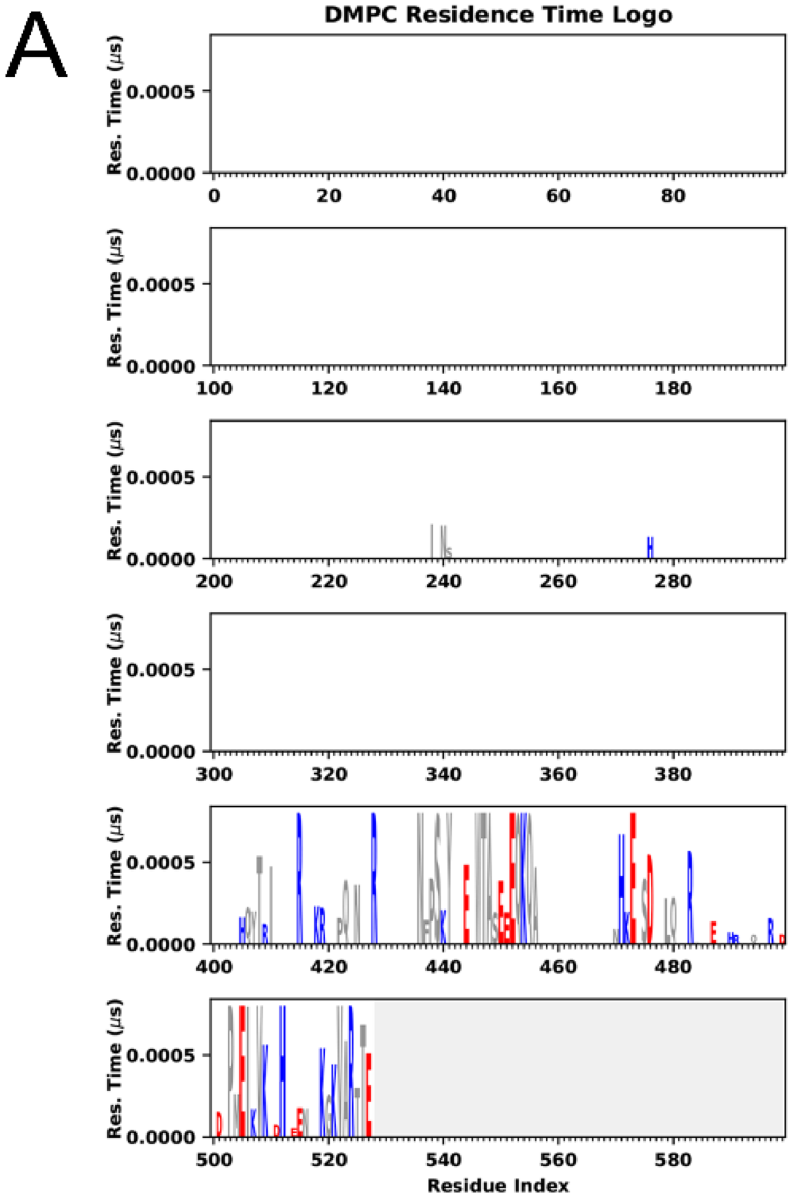

Figure S16A. Residence time for the DMPC lipids of the model membrane computed using the PyLipID Python package.[11, 12] The colors represent the charge of the amino acids (blue=positive, red=negative charge, gray=uncharged).

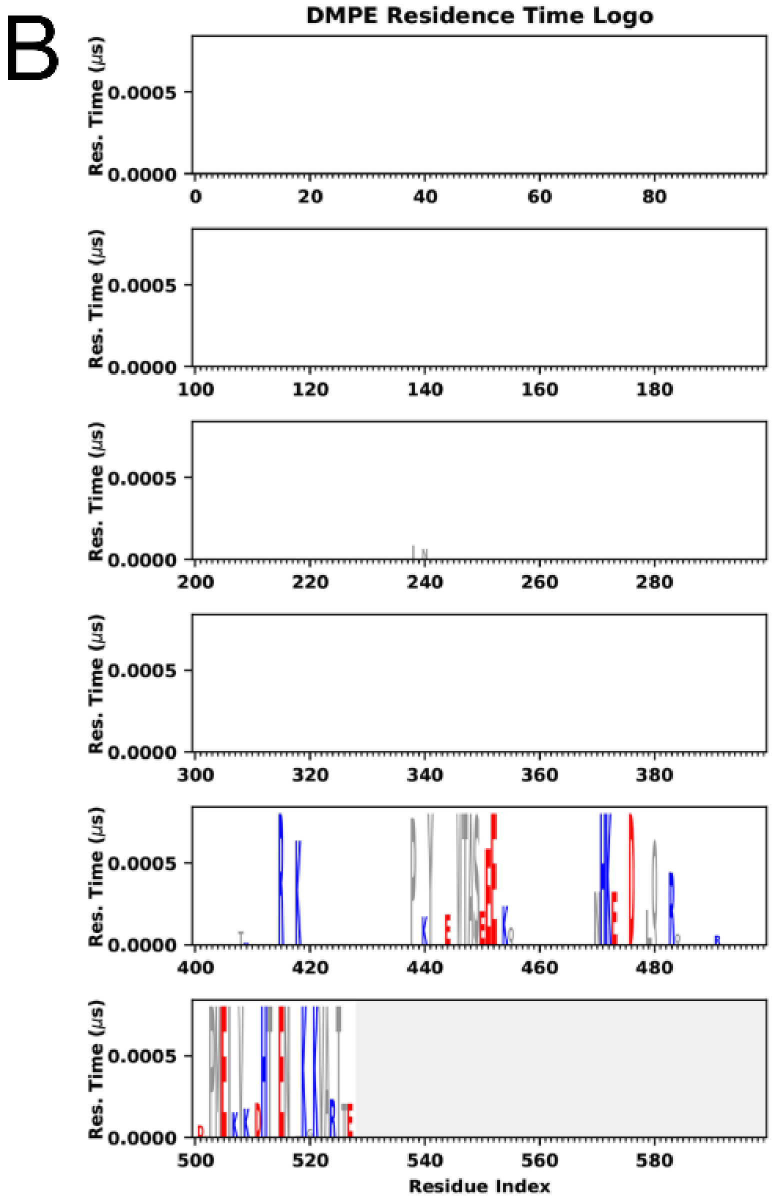

Figure S16B. Residence time for the DMPE lipids of the model membrane computed using the PyLipID Python package.[11, 12] The colors represent the charge of the amino acids (blue=positive, red=negative charge, gray=uncharged).

C

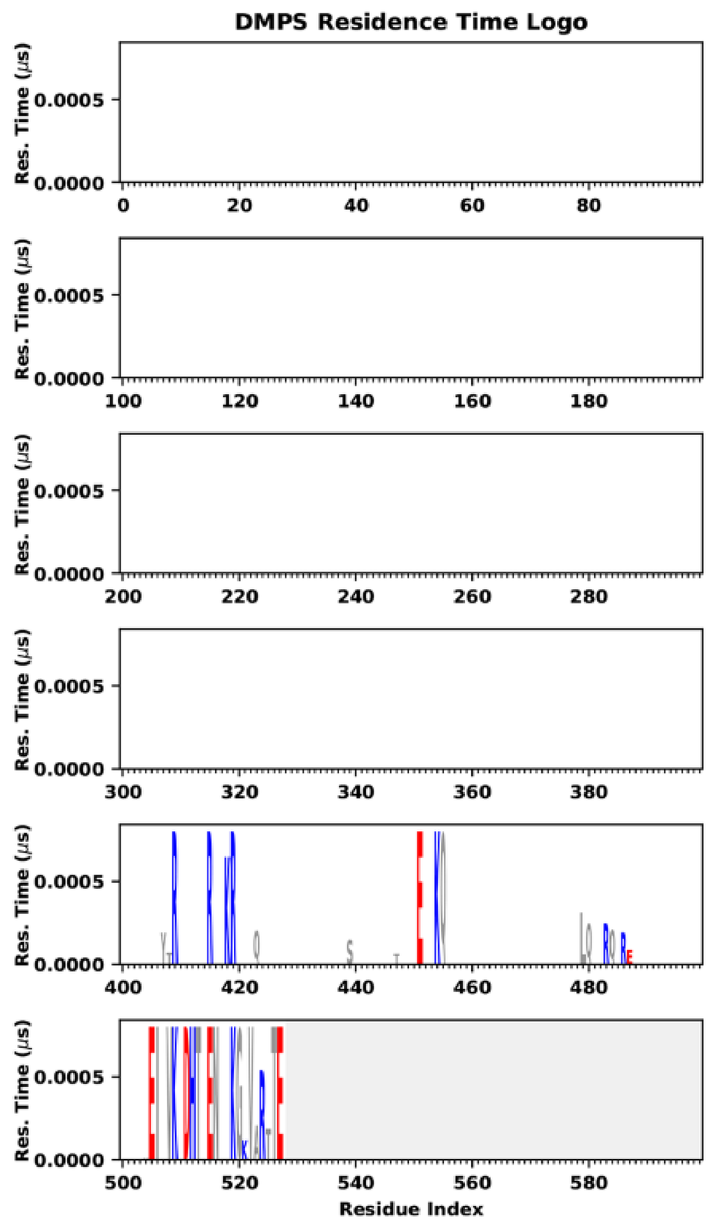

Figure S16C. Residence time for the DMPS lipids of the model membrane computed using the PyLipID Python package.[11, 12] The colors represent the charge of the amino acids (blue=positive, red=negative charge, gray=uncharged).

S19. Hydrogen bond analysis in the MD simulations

Table S3. Hydrogen bond analysis between the protein and the upper leaflet of the membrane. The limiting donor-acceptor distance was set to 3.0 Å and hydrogen bonds with the angleless than 20° were considered. Coloration of cells and amino acid names indicates charge (red=negative, blue=positive), while green color represents DMPC phospholipid. Analysis was done with HBonds Plugin in VMD.[11]

	Ori1-noMg(1)			Ori1-noMg(2)		
	Donor	Acceptor	Occupancy (%)	Donor	Acceptor	Occupancy (%)
1	DMPC	GLU451	0.00	ARG415	DMPS	44.76
2	GLN484	DMPS	0.00	ARG510	DMPS	38.69
3				ARG415	DMPS	32.76
4				ARG497	DMPS	32.06
5				LYS472	DMPE	14.47
6				DMPE	GLU505	13.66
7				LYS440	DMPE	12.85
8				LYS509	DMPS	12.83
9				ARG491	DMPS	12.61
10				LYS509	DMPC	11.54

	Ori1+Mg(1)			Ori1+Mg(2)		
	Donor	Acceptor	Occupancy (%)	Donor	Acceptor	Occupancy (%)
1	ARG409	DMPS	37.46	DMPE	GLU527	25.06
2	ARG483	DMPS	17.32	ARG524	DMPC	23.04
3	LYS440	DMPS	15.52	ARG524	DMPC	22.58
4	ARG524	DMPS	12.46	ARG524	DMPC	19.61
5	LYS509	DMPS	11.43	ARG524	DMPS	18.42
6	LYS521	DMPS	10.87	LYS521	DMPS	18.18
7	ARG510	DMPS	9.34	LYS440	DMPC	17.43
8	LYS509	DMPE	9.15	ARG415	DMPS	13.59
9	DMPE	GLU487	8.24	LYS521	DMPE	13.47
10	DMPS	GLU505	7.50	ARG524	DMPC	11.53

References

(1) Johnston, D.; Hudson, R. A. Phospholipids of the cone-rich chicken retina and its photoreceptor outer segment membranes. *Biochim.Biophys. Acta* **1974**, *369*, 269-277.
(2) Anderson, R. E.; Maude, M. B. Phospholipids of bovine rod outer segments. *Biochemistry* **1970**, *9*, 3624-3628.
(3) Lange, C.; Koch, K. W. Calcium-dependent binding of recoverin to membranes monitored by surface plasmon resonance spectroscopy in real time. *Biochemistry* **1997**, *36*, 12019–12026.
(4) Chang, S. H.; Chen, L. Y.; Chen, W. Y. The effects of denaturants on protein conformation and behavior at air/solution interface. *Colloids Surf. B* **2005**, *41*, 1–6.
(5) Barth, A. Infrared spectroscopy of proteins. *Biochim.Biophys. Acta* **2007**, *1767* (9), 1073-1101.
(6) De Meutter, J.; Goormaghtigh, E. Searching for a better match between protein secondary structure definitions and protein FTIR spectra. *Anal.Chem.* **2021**, *93*, 1561−1568.
(7) Tatulian, S. A. Structural characterization of membrane proteins and peptides by FTIR and ATR-FTIR spectroscopy. In *Lipid-protein interactions. Methods and protocols*, Kleinschmidt, J. H. Ed.; Methods in molecular biology, Springer, 2013; pp 177-218.
(8) Brand, I. *Application of Polarization Modulation Infrared Reflection Absorption Spectroscopy in Electrochemistry*; Springer Nature, 2020.
(9) Stephani, J. C.; Gerhards, L.; Khairalla, B.; Solov'yov, I. A.; Brand, I. How do antimicrobial peptides interact with the outer membrane of Gram-negative bacteria? Role of lipopolysaccharides in peptide binding, anchoring, and penetration. *ACS Infect. Dis.* **2024**, *10*, 763−778.
(10) Brendel, V.; Bucher, P.; Nourbakhsh, I. R.; Blaisdell, B. E.; Karlin, S. Methods and algorithms for statistical analysis of protein sequence. *Proc. Natl. Acad. Sci.* **1992**, *89*, 2002-2006.
(11) Humphrey, W.; Dalke, A.; Schulten, K. VMD: visual molecular dynamics. *J. Mol. Graph.* **1996**, *14*, 33-38.
(12) Song, W.; Corey, R. A.; Ansell, T. B.; Cassidy, C. K.; Horrell, M. R.; Duncan, A. L.; Stansfeld, P. J.; Sansom, M. S. PyLipID: A Python package for analysis of protein–lipid interactions from molecular dynamics simulations. *J. Chem. Theory Comput.* **2022**, *18*, 1188-1201.
